# A molecular dual carriageway

**DOI:** 10.7554/eLife.61148

**Published:** 2020-08-25

**Authors:** William J Allen, Ian Collinson

**Affiliations:** School of Biochemistry, University of BristolBristolUnited Kingdom

**Keywords:** nitrosomonas europaea, ammonium transporter, rhesus protein, transport selectivity, SSME, *Saccharomyces cerevisiae*, *E. coli*

## Abstract

In order to enter a cell, an ammonium ion must first dissociate to form an ammonia molecule and a hydrogen ion (a proton), which then pass through the cell membrane separately and recombine inside.

**Related research article** Williamson G, Tamburrino G, Bizior A, Boeckstaens M, Dias Mirandela G, Bage M, Pisliakov A, Ives CM, Terras E, Hoskisson PA, Marini AM, Zachariae U, Javelle A. 2020. A two-lane mechanism for selective biological ammonium transport. *eLife* **9**:e57183. doi: 10.7554/eLife.57183

Ammonium is an important metabolite for all forms of life. It acts as a major source of nitrogen for plants, fungi and bacteria, while in animals, it is used to maintain the pH balance inside cells (and is often produced as a toxic by-product of cellular processes). Each cell is surrounded by a membrane that is impermeable to charged particles like ammonium, so the uptake or release of such molecules requires specialized transporter proteins embedded in the membrane.

Following the pioneering work of biochemists and biophysicists, notably Peter Mitchell and Ronald Kaback, and a wealth of atomic structures revealed by X-ray crystallography and electron cryo-microscopy, the dynamic and biochemical actions of many biological transporters are well understood. So much so, it is rare nowadays to encounter a surprising new mechanism underlying solute transport. Now, in eLife, Ulrich Zachariae, Arnaud Javelle and colleagues at the University of Dundee, University of Strathclyde and Université Libre de Bruxelles, report just such a new mechanism for proton-driven ammonium transport (Williamson et al., 2020).

Even though the first atomic structure of an ammonium transporter was published 16 years ago, exactly how ammonium is transported could not be agreed on (Khademi et al., 2004). To cross the cell membrane, ammonium must pass through the centre of the transporter protein, which is very hydrophobic and therefore unfavourable to the passage of charged molecules. This led to the proposal that ammonium (with the chemical formula NH_4_^+^) dissociates into ammonia (NH_3_) and a positively charged hydrogen ion (H^+^), with only uncharged NH_3_ traversing the membrane (Khademi et al., 2004). The importance of this deprotonationevent is supported by other experimental evidence (Ariz et al., 2018). Yet, other studies have shown that for many ammonium transporters, movement of NH_3_ across the membrane is electrogenic, i.e., accompanied by a flow of positive charge generating an electric current (Ludewig et al., 2002).

The team, which includes Gordon Williamson, Giulia Tamburrino, Adriana Bizior and Mélanie Boeckstaensas joint first authors, reconcile these observations into a single, convincing model by showing that ammonium is first deprotonated on one side of the membrane, and that NH_3_ and H^+^ both travel across separately, whereupon they reunite to reform ammonium (Figure 1). Williamson et al. used detailed computer simulations to generate predictions for how transport is achieved, which were then validated in functional and electrophysiological analyses. As such, this work is an excellent example of how integrating computational and empirical observations can produce insights that would otherwise be very difficult to attain.

**Figure 1.**
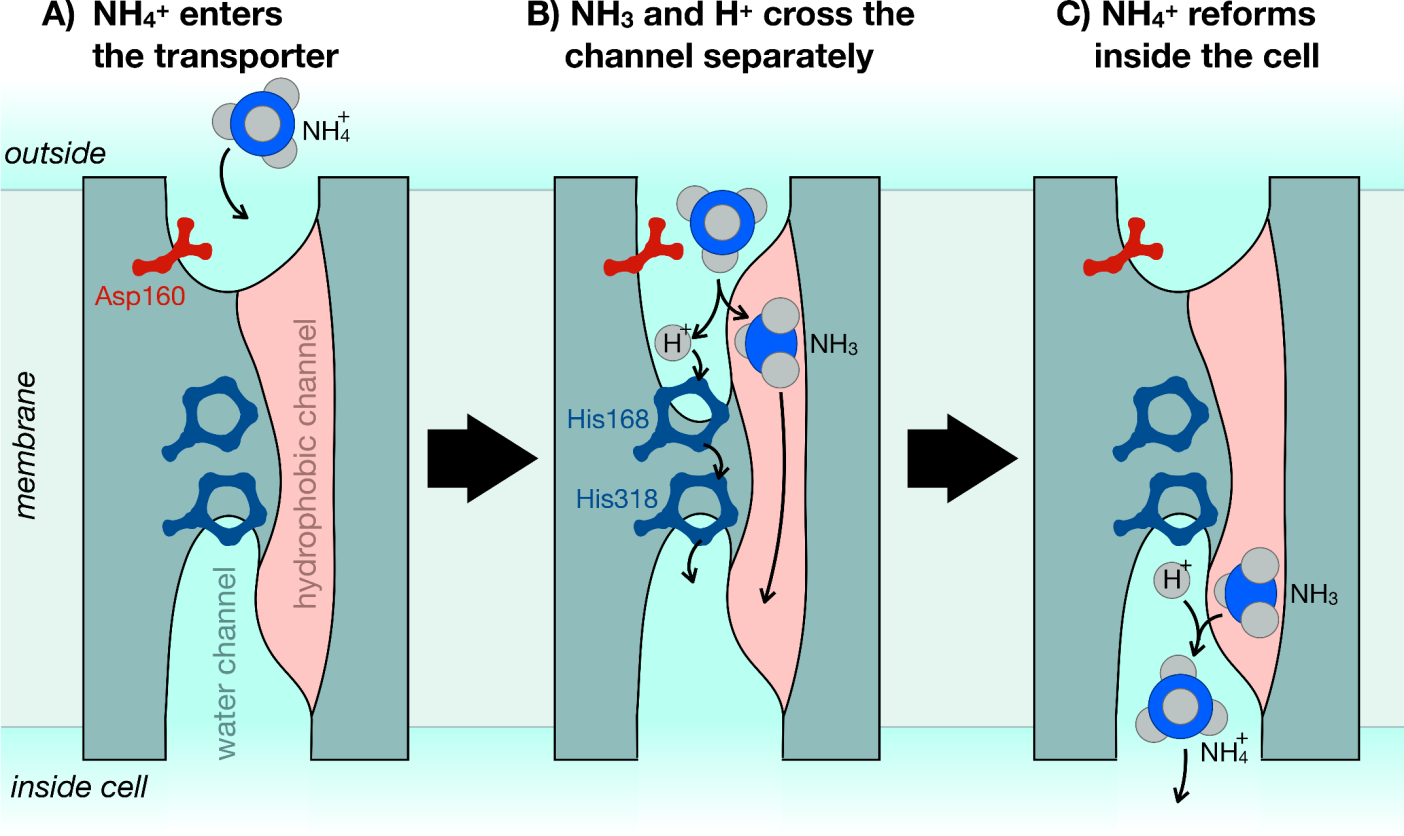
The mechanism of ammonium transporters. Ammonium is an important metabolite for many organisms. Until now, it was unclear how exactly ammonium is transported in and out of cells. Williamson et al. demonstrated that ammonium (NH_4_^+^) dissociates into ammonia gas (NH_3_) and a positively charged hydrogen (H^+^), which then traverse the cell membrane separately and re-join at the other end. (**A**) The ammonium transporter (teal) spans the whole membrane (grey). The two ends are open to the water-filled environment (cyan), but the only open passage through the centre is strongly water-repellent (pink). The entrance to the water channel has a pocket that specifically recognizes ammonium. (**B**) Ammonium binds into the pocket near the amino acid Asp160, which is essential to trigger the opening of the channel. The environment inside the pocket also allows ammonium to separate into NH_3_ and H^+^. NH_3_ can now cross the membrane through the hydrophobic channel (pink), and H^+^ crosses separately via the bridge formed of two copies of the amino acid histidine (His168 and His318). (**C**) Once inside the cell, NH_3_ and H^+^ recombine to make ammonium (NH_4_^+^).

A critical feature of any metabolite transporter is that it does not accidentally transport other molecules. This is a particular danger for potassium ions (K^+^), which are a similar size and shape to ammonium, but also for protons, which must cross the membrane as part of the transport reaction. The concentrations of these ions are tightly regulated inside cells, so even small leaks of either would be detrimental to most cells. The results of Williamson et al. reveal just how this selectivity is achieved. For example, the water-filled channel through which H^+^ moves is only formed when ammonium binds at the entrance to the transporter. The formation of the channel depends on a single amino acid, which is present in all known ammonium transporters: even a conservative amino acid substitution at this site completely stops the transporter from working (Figure 1A).

To prevent any potassium from leaking through, the fully formed water channel is blocked at its centre by two copies of the amino acid histidine (Figure 1B). Histidine readily takes up and releases protons at physiological (slightly basic) pH values (pH 7.4), so H^+^ can hop across the histidine bridge with relative ease. But the twin histidines block anything larger from getting through. If both of them are removed, the transporter becomes an open channel, letting both protonated ammonium and potassium flow through freely (Williamson et al., 2020).

Ammonium transporters share a similar three-dimensional structure, even in organisms with distant evolutionary origins. But it remains to be seen how widespread this specific transport mechanism is: do most ammonium transporters work in the same way, or are there some that transport NH_3_ while leaving the proton behind? And what structural features determine this? Also, further research is needed to determine how exactly the channel forms, how the proton is removed from the ammonium, and how the speed at which ammonium crosses the membrane is regulated. The breakthrough by Williamson et al. opens the door to these questions that could lead towards a complete understanding of a fundamental biological process widespread throughout nature.

Ammonium transporters are relevant to numerous, disparate scientific fields. For example, the central role ammonium plays in the global nitrogen cycle makes them important both for improving crop yields and for the breakdown of pollutants (Li et al., 2017; Fowler et al., 2013). Human ammonium transporters are essential for kidney function, and mutations have been implicated in various diseases (Kurtz, 2018; Huang and Ye, 2010). For those interested in other membrane transport processes, the idea of using selective protonation or deprotonation of small, ionisable solutes, or even the amino acid side chains of larger protein molecules, is compelling (Jiang et al., 2015; Wiechert and Beitz, 2017).
